# Saying No in Aesthetic Surgery: Ethical Framework for Declining High-Stakes Requests

**DOI:** 10.1007/s00266-026-05682-w

**Published:** 2026-02-19

**Authors:** Or Friedman, Yuval Paniri

**Affiliations:** 1 Head of Research and Development, Mayanei HaYeshua Medical Centre, Affiliated with the Gray Faculty of Medical & Health Sciences, Tel Aviv University, Tel Aviv, Israel; 2 The Dina Recanati School of Medicine, Reichman University, Herzliya, Israel; 3 Gray Faculty of Medical & Health Sciences, Tel Aviv University, Tel Aviv, Israel

**Keywords:** Aesthetic surgery, Medical ethics, Informed consent, Patient selection, Body dysmorphic disorder, Professionalism

## Abstract

**Background:**

Unlike criminal defense with its cab-rank duty, surgeons are constrained by nonmaleficence. This review examines when declining aesthetic surgery requests constitutes ethical care rather than prejudice.

**Objectives:**

To identify professional standards and empirical evidence guiding ethical decision-making when surgeons consider declining aesthetic surgery requests, and to provide a framework for principled refusal.

**Methods:**

Narrative review of professional standards (GMC, RCS, ASPS, ISAPS), outcome data on higher-risk procedures, comparative analysis of jurisdictional safeguards, and ethics literature from plastic surgery. PubMed searches used terms: [(aesthetic surgery OR cosmetic surgery) AND (ethics OR informed consent OR patient selection OR refusal OR body dysmorphic disorder)] for 2014–2025, supplemented by professional society websites.

**Results:**

Global demand reached 38 million aesthetic procedures in 2024. Ethics discourse remains underrepresented (approximately one in 1000 articles), with autonomy disproportionately emphasized over beneficence, nonmaleficence, and justice. Abdominoplasty complications cluster around 2–4%; gluteal fat grafting carries elevated mortality. Modifiable risks include nicotine, cannabis, and GLP1 therapy. Body dysmorphic disorder prevalence approaches 18.6% among candidates. Contemporary guidance emphasizes surgeon-led consent, cooling-off periods, psychological screening, and discretion to decline when benefit is doubtful. Ethically defensible refusal requires articulable clinical reasons, documentation, and alternatives—never moral disapproval.

**Conclusions:**

Structured frameworks applying the four bioethical principles—autonomy, beneficence, nonmaleficence, and justice—provide actionable guidance. Principled refusal, when clinically indicated and compassionately explained, is not failure but expression of professionalism.

**Level of Evidence V:**

This journal requires that authors assign a level of evidence to each article. For a full description of these evidence-based medicine ratings, please refer to the Table of Contents or the online Instructions to Authors www.springer.com/00266.

## Introduction

Aesthetic surgeons are custodians of bodily integrity. In elective surgery, the threshold question is whether the proposed operation is clinically indicated, proportionate, safe, and truly consented to under conditions making autonomy meaningful. Rather than labeling patients as “difficult” or “undesirable,” we focus on ethically salient features of requests: unrealistic expectations, distorted risk perception, external pressures, unstable health behaviors, or procedures whose harm profile overwhelms plausible benefit. Surgery maintains a primary duty to patient welfare, including the duty not to harm [1–4].

ISAPS reports approximately 38 million procedures in 2024 [5]. Yet ethical discourse remains underrepresented in plastic surgery literature. A 2021 systematic review found that among more than 100,000 articles, only a small fraction focused on ethical principles, with autonomy receiving disproportionate emphasis relative to beneficence, nonmaleficence, and justice [6]. This gap persists despite calls for increased ethics education [7–9].

Throughout this review, we examine ethically high-stakes requests—situations where foreseeable benefit is doubtful, risk is disproportionate, consent is fragile, or continuity cannot be guaranteed.

## Methods

### Literature Identification

PubMed searches used terms: [(aesthetic surgery OR cosmetic surgery) AND (ethics OR informed consent OR patient selection OR refusal OR body dysmorphic disorder OR risk assessment)] for 2014–2025, supplemented by professional society websites (GMC, RCS, ASPS, ISAPS, BAAPS) and hand-searching references from systematic reviews [6–8, 10–12]. 

## Results

### The Ethics Gap in Aesthetic Surgery

Despite 38 million procedures in 2024 [5, 13], systematic analysis reveals marginal ethical discourse in plastic surgery scholarship. Among > 100,000 articles, fewer than 100 substantively addressed ethical principles, with autonomy invoked in ~ 70%, while beneficence, nonmaleficence, and justice received less attention [6].

### Evidence-Based Risk Stratification

*Procedure-specific risks* Gluteal fat grafting carries atypically high mortality risk, mandating subcutaneous-only injection and ultrasound guidance in some jurisdictions [14–16]. Abdominoplasty shows 2–4% major complication rates, rising with combined operations, higher BMI, and older age [17, 18].

*Patient-specific modifiable factors* Active smoking increases wound complications [19, 20]. Cannabis use associates with higher complication rates [21, 22]. GLP1 receptor agonists raise aspiration concerns; consensus statements advise medication holds or full-stomach management [23]. GLP1 users undergoing breast procedures show increased wound complications and lower nutritional markers [24, 25].

These modifiable risks transform refusal into pathway: Declining while supporting optimization (smoking cessation, medication timing, nutritional improvement) aligns beneficence with nonmaleficence [11].

### Contemporary Professional Standards (Table 1)

**Table 1:** Contemporary professional standards for ethical aesthetic surgery practice

Domain	Key requirements	Regulatory examples
Consent process	Operating surgeon must conduct consent conversation; cooling-off period required; consent revisited after reflection time	GMC [1], RCS [2], ASPS [3], ISAPS [4]
Psychological assessment	Screen for BDD and psychological vulnerability; refer for psychiatric assessment when indicated	ISAPS [4], ASPS [3]
Marketing and advertising	No misleading claims; ban on testimonials (some jurisdictions); restrictions on before/after imagery; no undue pressure or time-limited offers	GMC [1], Ontario CPSO [44], BC CPSBC [45]
Practitioner qualifications	Practice within competence; regulated environment; audit and oversight	GMC [1], RCS [2], Singapore MOH [42, 43]
Refusal discretion	May decline when benefit doubtful, risk disproportionate, or consent compromised; must document clinical reasons; must not discriminate	ASPS [3], ISAPS [4], GMC [1]
Medical tourism	Continuity of care planning required; aftercare arrangements essential; patient education on risks	BAAPS [34], RCS [35]

*GMC* General Medical Council (UK), *RCS* Royal College of Surgeons (UK), *ASPS* American Society of Plastic Surgeons, *ISAPS* International Society of Aesthetic Plastic Surgery, *CPSO* College of Physicians and Surgeons of Ontario, *CPSBC* College of Physicians and Surgeons of British Columbia, *MOH* Ministry of Health, *BAAPS* British Association of Aesthetic Plastic Surgeons

*Convergent standards across jurisdictions *(Table 1) emphasize surgeon-led consent, psychological vulnerability assessment, reflection time, advertising restrictions, and competence within regulated environments [1–4]. Applying Beauchamp and Childress’s four principles reveals that while autonomy dominates discourse, beneficence, nonmaleficence, and justice require equal consideration [11, 12].

These documents invite surgeons to decline when proceeding would compromise welfare, provided refusal is grounded in clinical reasons, free of bias, with explanation and referral. Contemporaneous documentation provides both ethical clarity and legal defensibility [26, 27].

### Psychological Vulnerability and Body Dysmorphic Disorder

BDD fundamentally alters risk-benefit calculus. Recent meta-analysis reports 18.6% prevalence among aesthetic surgery candidates [28]. Patients with BDD typically experience limited benefit and risk symptom worsening, as underlying distress remains unaddressed [29].Screening and psychiatric referral when BDD is suspected are essential safeguards honoring both nonmaleficence and beneficence, while ensuring consent is informed by realistic expectations.

### Market Pressure and Consent Quality

Consent quality is jeopardized by marketing, time-limited offers, and influencer culture. Contemporary guidance counters these with cooling-off periods, testimonial bans in some jurisdictions, and requirements that the operating surgeon secures consent [1, 2, 30, 31]. When surgery is marketed as commodity, the physician–patient relationship risks ethical erosion. Justice becomes implicated when commercial pressures create disparities: Resourced patients access careful deliberation while those seeking “discount” procedures encounter inadequate counseling [32].

## Discussion

### Ethically Complex Requests in Contemporary Practice

We avoid labeling patients; instead, we discern when requests reflect autonomous choice versus emergence from psychological vulnerability, commercial pressure, or distorted perceptions of benefit and risk. Most difficult consultations are challenging because risk and expectation are out of balance [12].

Consider contemporary complexity:

- Adolescents seeking change amid identity flux [33]
- Medical tourists without continuity guarantees [34, 35]
- Requests for outlier-mortality procedures [14–16]
- Smokers or cannabis users with elevated risks [19, 21, 22]
- Patients on GLP1 therapy [23]
- Revision-seekers convinced only repetition will satisfy

None of these makes a person “undesirable.” They change the ethical arithmetic of benefit, risk, and consent.

### Framework for Ethical Refusal (Table 2, Fig. 1)

**Table 2:** Framework for ethical refusal: applying the four principles

Principle	When refusal is indicated	Communication strategy	Alternative pathway
Nonmaleficence	Risk unusually high and cannot be mitigated Foreseeable harm outweighs benefit Patient factors substantially elevate complication risk Procedure carries elevated mortality	“The risks of this procedure in your current situation are higher than I can safely manage. Let me explain why...”	Risk factor optimization (smoking cessation, weight stabilization) Alternative lower-risk procedures Referral to center with specialized capability
Autonomy	Understanding/expectations make valid consent unlikely External pressures suggest choice not autonomous Evidence of BDD or conditions impairing realistic assessment	“I’m concerned that you may not have all the information you need to make this decision. Here’s what I’m seeing...”	Extended counseling period Psychiatric consultation Cooling-off period with follow-up consultation Independent second opinion
Beneficence	Benefit too small relative to risk Alternative approaches offer better prospects Optimization would improve outcomes	“I want to help you achieve your goals, but I think there’s a better way to get there...”	Non-surgical options Staged procedures Optimization period with clear reconsideration criteria Conservative alternatives
Justice	Environment cannot support procedure safely Continuity of care cannot be assured Technique safety profile contested	“I cannot provide you with the standard of care you deserve under these circumstances...”	Referral to appropriate facility Local follow-up arrangements before proceeding Wait for evidence/technique evolution

**Fig. 1:**
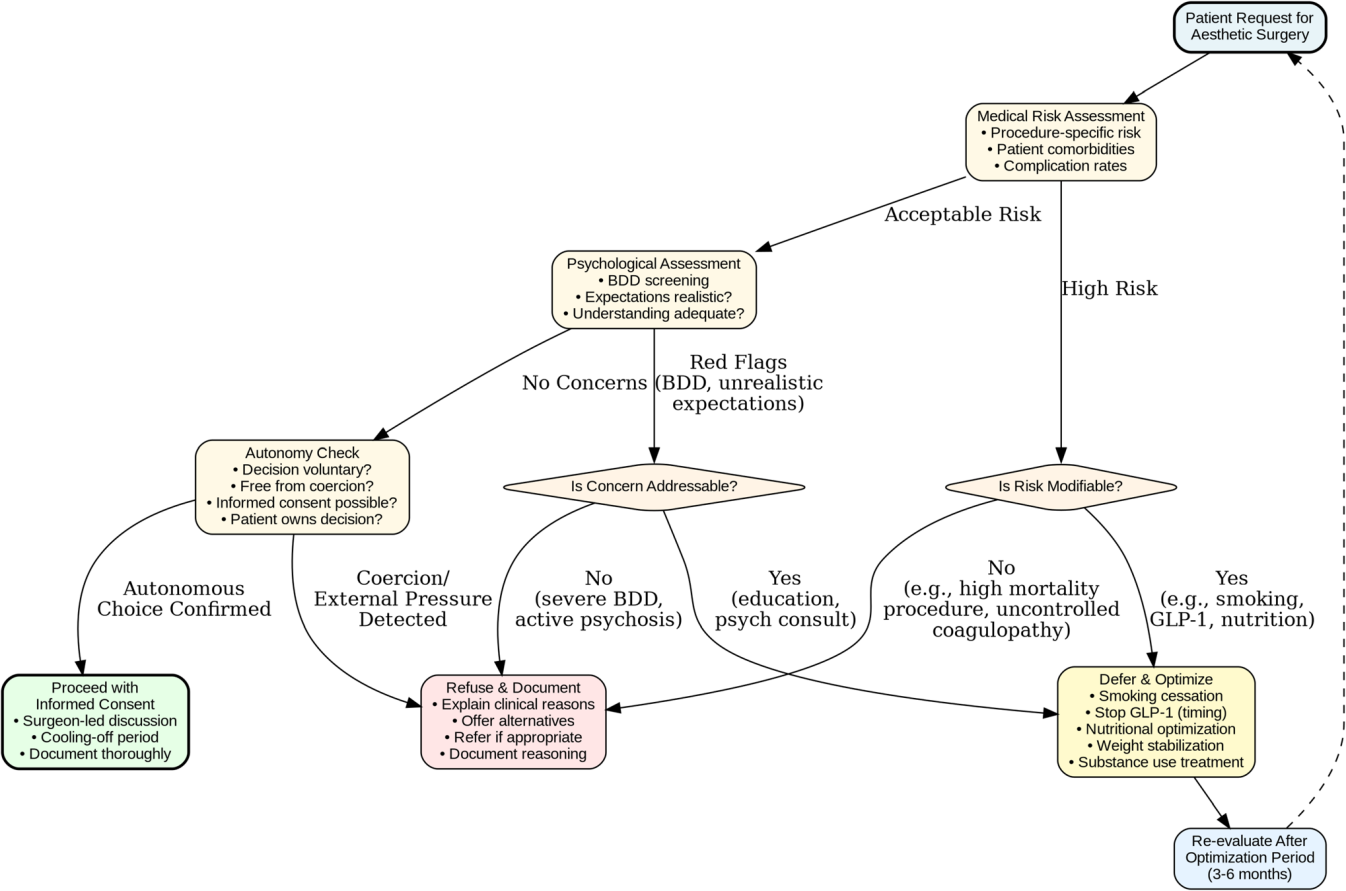
Ethical decision-making pathway for aesthetic surgery requests. A structured algorithm for evaluating ethically complex aesthetic surgery requests. The pathway distinguishes between refusal (for non-modifiable contraindications such as severe body dysmorphic disorder, coercion, or procedures with unacceptable risk profiles) and deferral with optimization (for modifiable risks including active smoking, cannabis use, GLP-1 therapy, or nutritional deficits). This framework operationalizes the four bioethical principles: nonmaleficence (risk assessment), autonomy (consent evaluation), beneficence (optimization opportunities), and justice (consistent application across cases). Red pathways indicate decline with referral; yellow pathways indicate defer and optimize; green pathway indicates proceed with informed consent. The dashed line represents the re-evaluation loop after optimization period (typically 3-6 months)

Defensible refusal rests on articulable reasons rooted in core ethical principles (Table 2) [11]: 

*Nonmaleficence* Risk unusually high, foreseeable harm outweighs benefit, patient factors elevate complications, procedure carries elevated mortality.

*Autonomy* Understanding/expectations make valid consent unlikely, external pressures suggest non-autonomous choice, evidence of BDD or conditions impairing realistic assessment.

*Beneficence* Benefit too small relative to risk, alternatives offer better prospects, optimization would improve outcomes.

*Justice* Environment cannot support procedure safely, continuity cannot be assured, technique safety profile contested.

### Communication and Process

When declining, the task is preserving dignity while making reasoning intelligible. This requires: stating reasons plainly, distinguishing person from request, acknowledging goals, and offering pathways—optimization, staged timing, alternatives, or referral.

Patients report that unexplained refusal feels paternalistic; reasoned explanation treating them as partners feels respectful [36]. Declining is not abandonment. It entails explaining reasoning, documenting comprehensively, offering alternatives with clear reconsideration criteria, and signposting to independent assessment [37]. Many academic centers now involve bioethicists and legal counsel in complex cases [38].

Surgeons must police their own biases. Dislike of lifestyle, politics, or social characteristics is not ethical grounds for refusal [1–4, 37]. The standard: could another surgeon, reading the note, see clinical rationale applying regardless of who sat in the chair?

This framework represents principlism applied to aesthetic practice: autonomy respected through explanation, nonmaleficence honored by declining harmful interventions, beneficence fulfilled by offering alternatives, justice served by consistent standards [11].

### The Legal Analogy and its Limits

The courtroom analogy is limited. In law, access to representation serves justice; in surgery, restraint serves nonmaleficence. The legal profession’s non-identification principle teaches: Surgeons should resist conflating patients’ traits with their entitlement to respectful engagement [11].But the divergence is decisive. Surgeons balance respect for autonomy with duty not to offer harm. Saying no, when reasons are sound and explained, manifests professionalism. [39, 40]

### Global Variation in Safeguards (Table 3)

**Table 3:** Global variation in aesthetic surgery safeguards

Jurisdiction	Key safeguards	Enforcement mechanism
UK (GMC, RCS)	Surgeon-led consent mandatory Cooling-off periods Marketing subject to professional standards Psychological screening emphasized	Professional regulation; fitness-to-practice proceedings; practice restrictions
Australia (ahpra/medical board)	GP referral required for cosmetic surgery Extended cooling-off for minors and high-risk procedures National guidelines for non-surgical cosmetics (2025) Age-based safeguards Influencer advertising restrictions	National registration standards; mandatory reporting; practice audits; sanctions including suspension
Singapore (MOH)	Liposuction as regulated service requiring accreditation Premises standards Training framework for aesthetic procedures Advertising controls	Licensing requirements; parliamentary oversight; advertising enforcement
Canada (provincial colleges)	Ontario: Ban on testimonials; evidentiary requirements for claims British Columbia: Restrictions on incentives, comparative claims, unqualified before/after imagery	College complaints process; practice reviews; professional discipline
United Arab Emirates / Dubai	Permits required for health advertising Penalties for unlicensed promotional content Restrictions on OR filming for advertising Social media content regulation	Prior authorization system; sanctions; DHA enforcement (Dubai)
USA(ASPS, state boards)	Code of ethics requirements Informed consent standards State-by-state medical board oversight Varies by jurisdiction	State medical board discipline; society membership requirements; voluntary compliance with society guidelines

*Ahpra* Australian Health Practitioner Regulation Agency, *DHA* Dubai Health Authority

#### The Ethical Vocabulary is Shared; Operational Guardrails Differ by Jurisdiction (Table 3).

UK guidance places surgeon-led consent and reflection time centrally, framing marketing as professional activity [1, 2]. Australia hardwires GP referral, mandates extended cooling-off for minors and high-risk procedures, and brings non-surgical practice under explicit guidelines effective 2025 [41]. UK societies issue cautions on cosmetic tourism [34, 35].

Singapore regulates liposuction via accreditation [42, 43]. Canadian provincial colleges prohibit testimonials (Ontario) and restrict incentives (British Columbia) [44, 45]. Gulf states require advertising permits and penalize unlicensed content [46, 47].

These convergences—surgeon-owned consent, reflection time, testimonial limits, stronger oversight—are ethically aligned despite differing legal levers. When conditions for safe care cannot be met, ethical restraint is warranted even if law permits operation elsewhere. Justice implicates international practice: traveling patients face information asymmetries, lack of recourse, and compromised revision access [12, 34]. 

### Implementation in Practice (Fig. 1)

Translating principles into habits aids practical application (Fig. 1):

- Ask whether operation confers benefit outweighing harm in patient’s real circumstances
- Ensure operating surgeon conducts consent and revisits after reflection
- Test for external pressures without pathologizing patient
- Attend to modifiable risks (nicotine, cannabis, GLP1, nutrition)
- Recognize when safety profile demands constraints or deferral
- When declining, explain reasons, record contemporaneously, offer alternatives

These steps are modest and narrative rather than algorithmic, but reproducible and auditable—what professionalism requires. Simple checks like ensuring adequate albumin in massive-weight-loss populations can reduce wound problems and make deferral ethically and clinically compelling when levels are low [25]. This represents integration of ethical reasoning with clinical risk assessment.

The paucity of formal ethics education in training [7, 8] suggests many practitioners navigate challenges without structured frameworks. Academic institutions have an obligation to formalize ethics education using case-based learning, principlist frameworks, and communication skills development. The goal is cultivating ethical reflexivity: capacity to recognize ethical dimensions, analyze systematically, and act with conviction and humility [9].

## Conclusions

The profession needs disciplined fidelity to existing standards, honest use of empirical risk, humility about uncertainty, and courage to decline when benefit cannot outweigh harm. The legal analogy teaches non-moralization; the divergence from law affirms abstention is sometimes the ethical act.

The underrepresentation of ethics discourse reflects a missed opportunity: every declined request, every difficult conversation, every tension between commercial pressure and professional obligation represents a case study in applied ethics [6, 9].

The framework proposed—grounding decisions in the four principles of autonomy, beneficence, nonmaleficence, and justice; ensuring refusals are articulable, documented, bias-free, and explained—is application of long-established ethical theory to aesthetic practice’s specific challenges [11]. What is required is not new ethics but renewed commitment: Recognition that saying no, when clinically indicated and compassionately explained, is not failure but expression of professionalism at its most fundamental.

## Declarations

### Meeting Presentation

This work has not been presented at any meeting.
